# Supplementary material for: Epigenetic landscape of pancreatic neuroendocrine tumours reveals distinct cells of origin and means of tumour progression
Source: Commun Biol. 2020 Dec 7;3:740. doi: 10.1038/s42003-020-01479-y (PMC7721725; doi:10.1038/s42003-020-01479-y)
Supplement: Supplementary file 1 — Supplementary Information [file 42003_2020_1479_MOESM1_ESM.pdf]

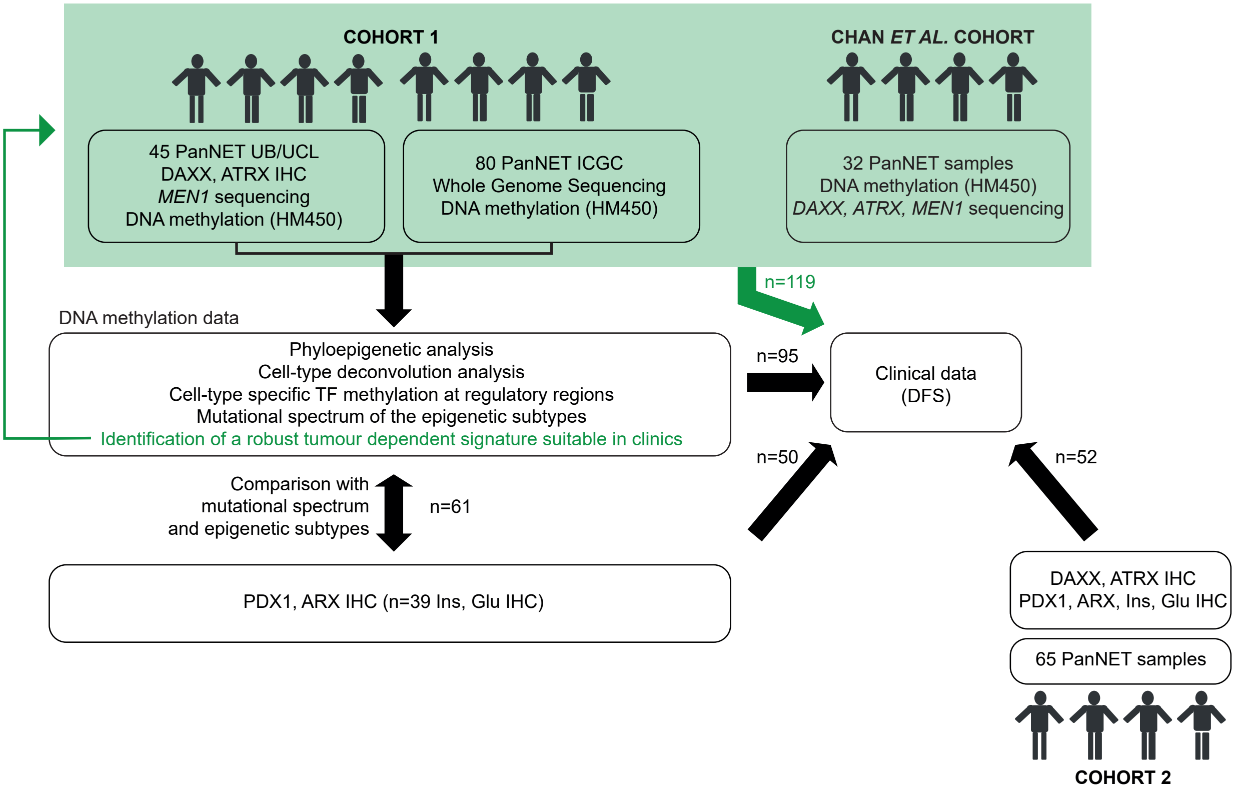

Supplementary Fig. 1. Flow-chart indicating number of patients used for each analysis.

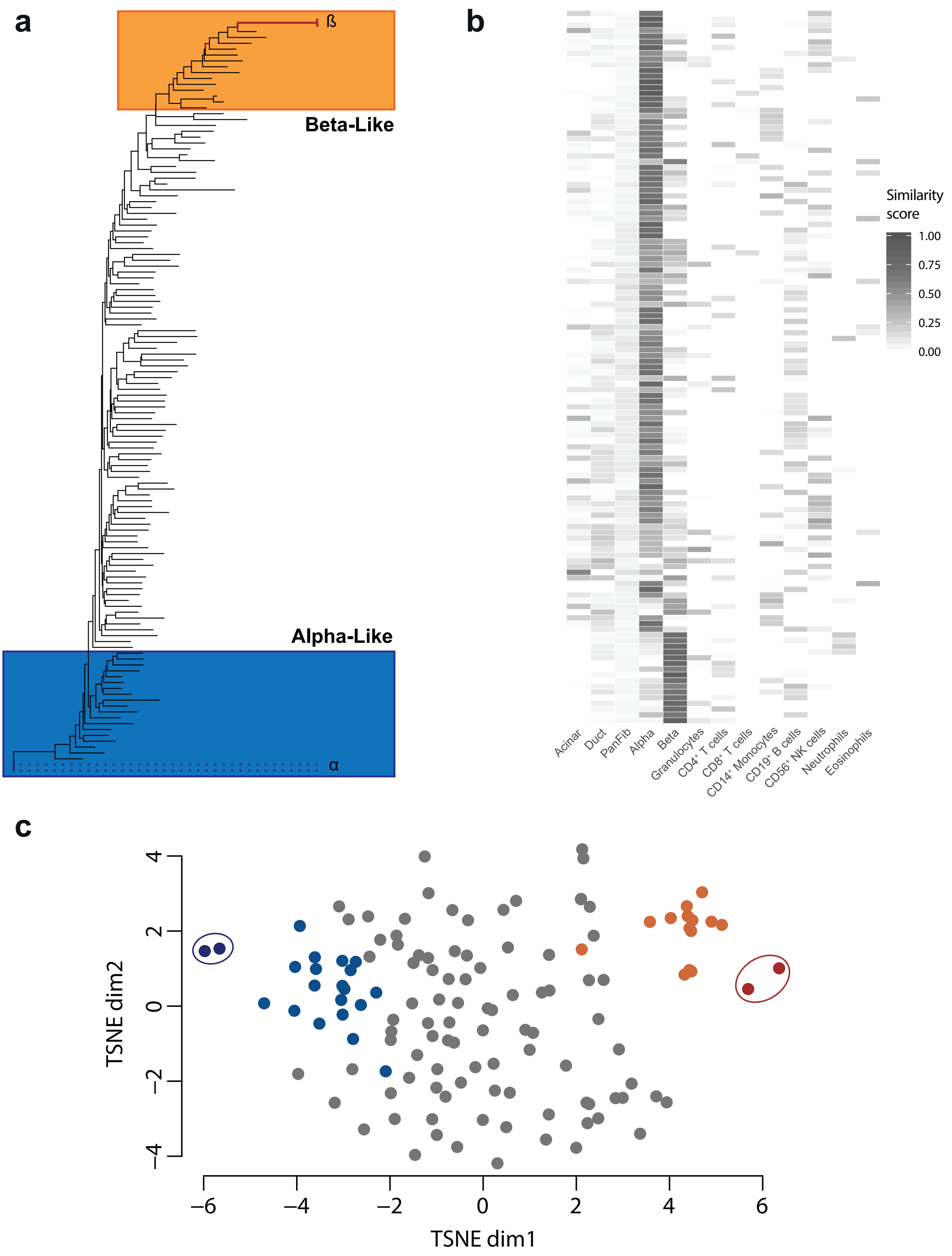

Supplementary Fig. 2. a. Phyloepigenetic analysis of PanNET and normal α- and β-cell samples. Rooted tree was created with an arbitrary chosen α-sample as the root and according to the differentially methylated CpGs between sorted normal α- and β-cell samples (n=2131, adj. p-value<0.001 and |Δβ|>0.2). In the blue and orange squa-res are included the α-like and β-like tumours, respectively. **b.** Cell type contributions of sorted exocrine (acinar, duct, pancreatic fibroblasts) and endocrine (α- and β-cells) pancreatic cells and blood cells (granulocy-tes, CD4+ and CD8+ T cells, CD14+ Monocytes, CD19+ B cells, CD56+ natural killer cells, neutrophils and eosinophils cells), in each PanNET sample based on methylation profiles. Scale refers to the percentage of contribution of each sorted cell type to the tumors normalized to one. Samples are sorted according to figure 1a **c.** t-SNE plot depicting PanNET and normal α- and β-cell samples. In the blue and red circles are indicated α- and β-cell samples, respectively (2 samples for each cell type). Orange and blue dots indicate α-like and β-like tumours, respectively.

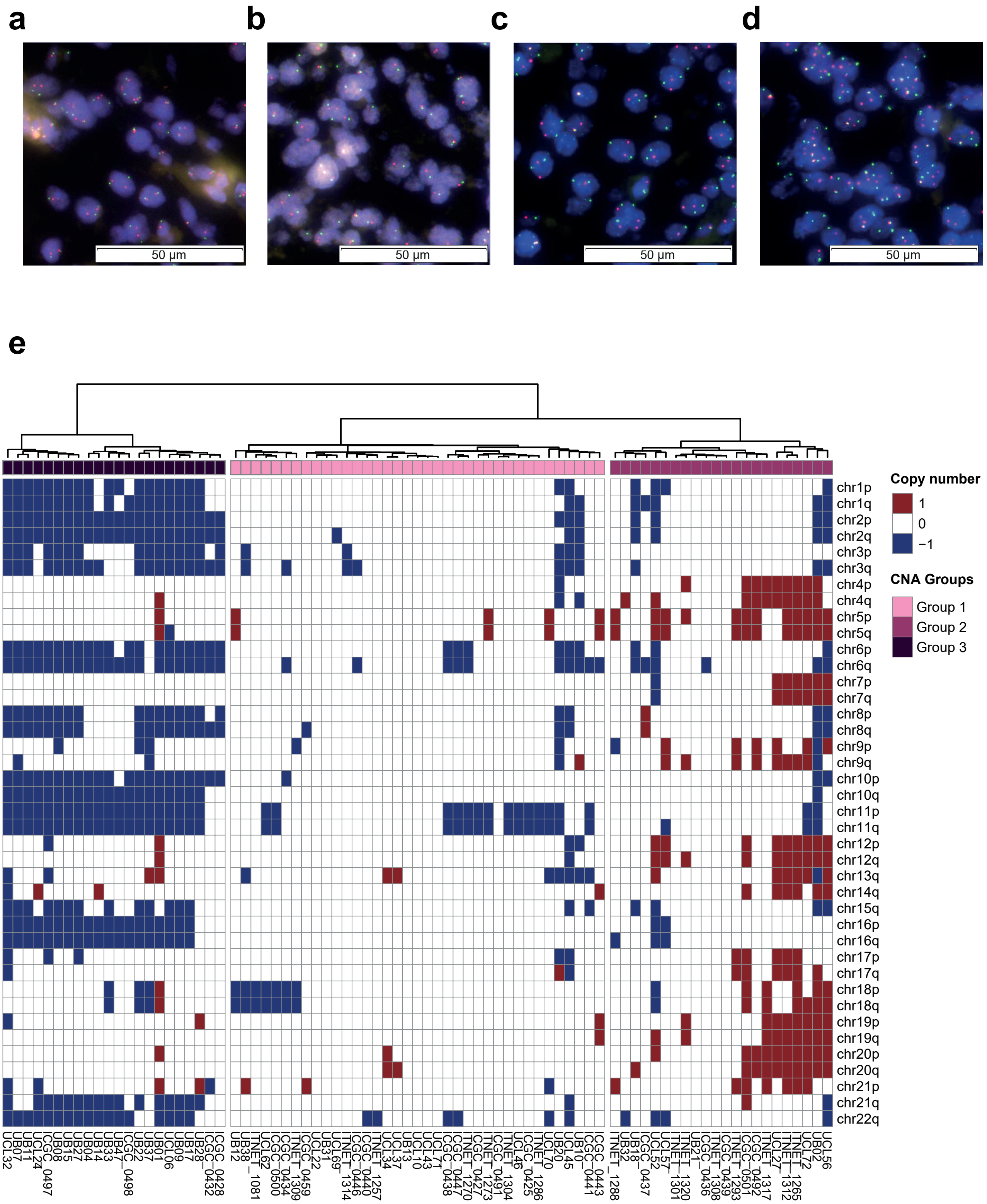

Supplementary Fig. 3. In fig. **a** and **b** illustrative pictures for Cenp11 (green) and *MEN1* (red) FISH. In fig. **c** and **d** illustrative pictures for Cenp17 (red) and Cenp4 (green) FISH. **e.** Dominant inferred copy number (CN) for each chromosomal arm in each tumour. Chromosome arms in rows and patients in columns (red: amplification, blue: deletions, cut-off at ±0.2). CNA group 1 with limited number of events; group 2 with losses and gains; group 3 with recurrent chromosomal losses.

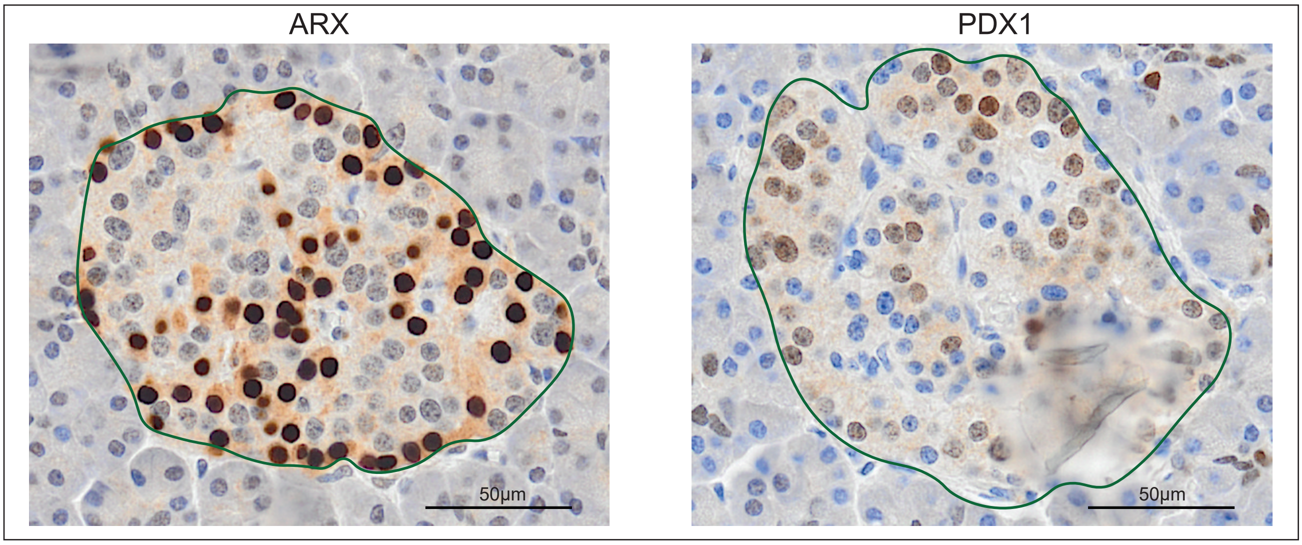

Supplementary Fig. 4. ARX and PDX1 IHC mark respectively α- and β-cells in normal pancreatic islets (drawn in green). Many exocrine and ductal cells present positivity for PDX1, as known (Jennings et al., 2015, 2013; Larsen and Grapin-Botton, 2017; Segerstolpe et al., 2016).

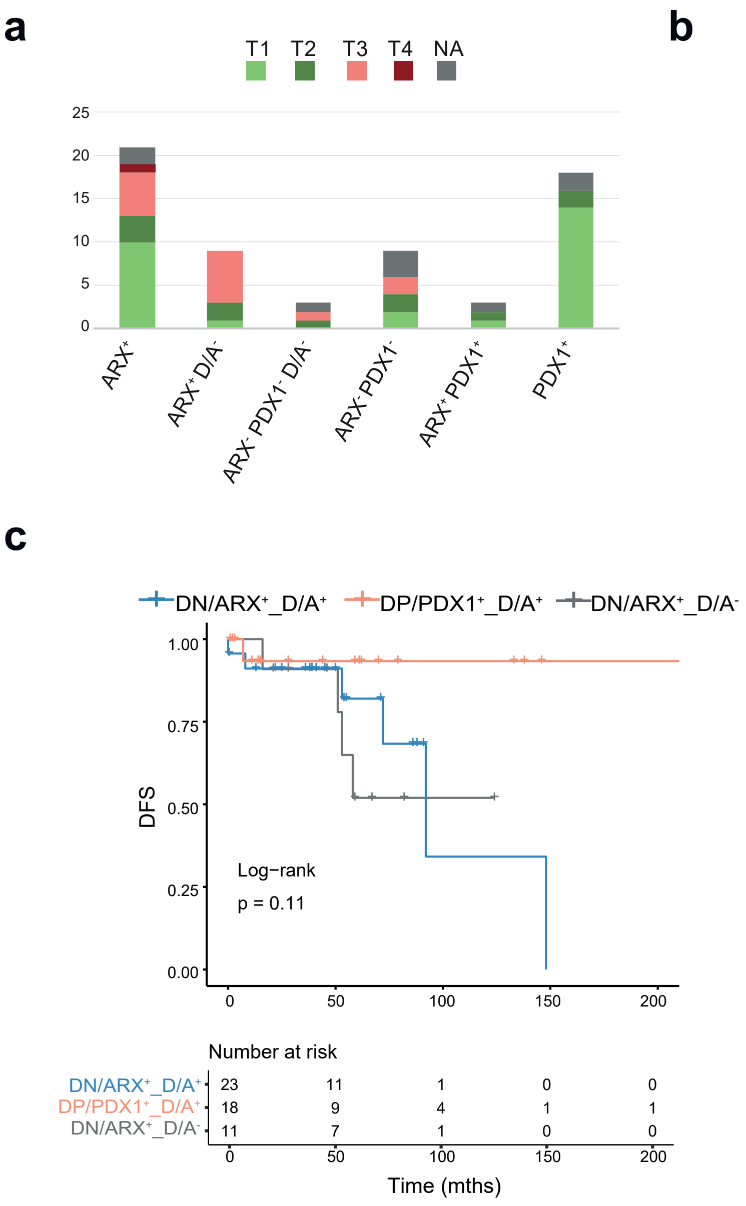

Supplementary Fig. 5. a. Number of PanNETs (y-axis) per cell-type subtype (x-axis) and relative to tumour stage **b.** Number of PanNETs (y-axis) per cell-type subtype (x-axis) and relative to patient relapse. **c.** Kaplan-Meier disease free survival of 52 patients (cohort 2) stratified according to PDX1, ARX and DAXX/ATRX IHC.

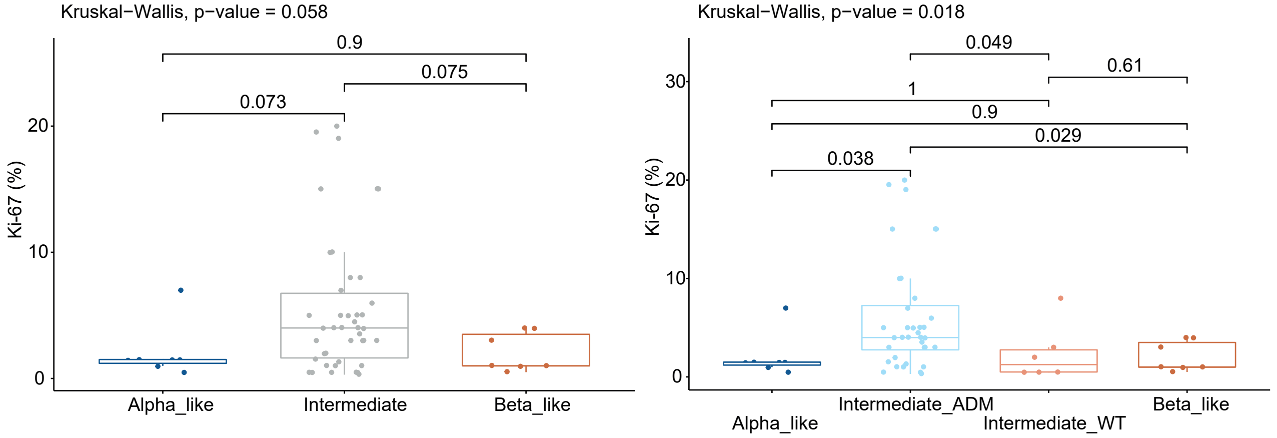

Supplementary Fig. 6. Boxplots of Ki-67 values (%) per epigenetically defined tumour groups. Each dot represents one PanNET patient. Only intermediate-ADM tumours showed significantly higher Ki-67 values (%) when compared to the other tumour subtypes. We tested for significance using Wilcoxon-Mann-Whitney-test (*U* test) when comparing two groups and Kruskal-Wallis-test (H-test) when comparing more than two groups.

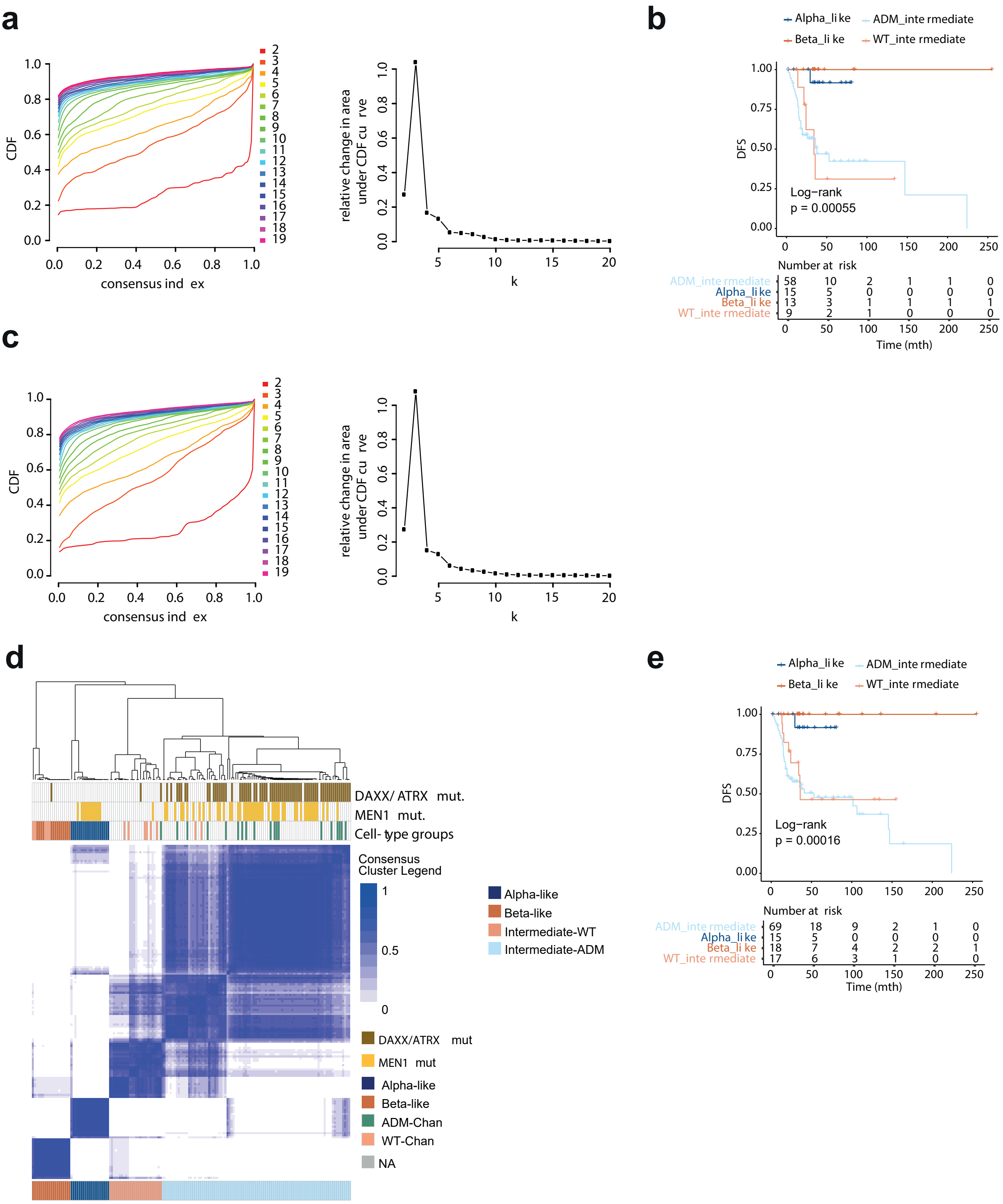

Supplementary Fig. 7. a. Plot of cumulative distribution functions (CDF) for the consensus matrix for each k (left) and relati-ve change in area under the CDF curve (right). Consensus clustering was performed for the 125 PanNETs (cohort 1) according to the 6364 differentially methylated sites between α-like, β-like and intermediate tumours (adj. p-value<0.001 and |Δβ|>0.2). **b.** Kaplan-Meier disease free survival of the 95 patients (cohort 1) stratified according the consensus clustering groups (α-like, β-like, intermediate-WT, intermediate-ADM). **c.** Plot of cumu-lative distribution functions (CDF) for the consensus matrix for each k (left) and relative change in area under the CDF curve (right). Consensus clustering was performed for the 157 PanNETs (cohort 1 and Chan et al. cohort) according to the 6359 differentially methylated sites (5 sites were excluded after filtering and normaliza-tion processes) identified from the analysis of cohort 1. In **d** the relative consensus clustering matrix for k=4. Each column represents a patient. Consensus cluster correlation is indicated according to the blue scale as indicated. **e.** Kaplan-Meier disease free survival of the 119 patients (cohort 1 and Chan et al. cohort) stratified according the consensus cluster groups (α-like, β-like, intermediate-WT, intermediate-ADM).
