# Supplementary material for: Epigenetic landscape of pancreatic neuroendocrine tumours reveals distinct cells of origin and means of tumour progression
Source: Commun Biol. 2020 Dec 7;3:740. doi: 10.1038/s42003-020-01479-y (PMC7721725; doi:10.1038/s42003-020-01479-y)
Supplement: Supplementary file 2 — Description of Additional Supplementary Files [file 42003_2020_1479_MOESM2_ESM.pdf]

Description of Additional Supplementary Files

File Name: Supplementary Data 1
Description: Clinico-pathological and molecular characteristics of PanNET cohort 1

File Name: Supplementary Data 2
Description: Differentially methylated CpG sites identified comparing normal α-to normal β-cells

File Name: Supplementary Data 3
Description: Alpha specific TF checkpoint CpG sites located at normal islet regulatory regions

File Name: Supplementary Data 4
Description: Beta specific TF checkpoint CpG sites located at normal islet regulatory regions

File Name: Supplementary Data 5
Description: Clinico-pathological and molecular characteristic of cohort 2

File Name: Supplementary Data 6
Description: Differentially methylated CpG sites identified comparing α-like to β-like PanNETs

File Name: Supplementary Data 7
Description: Differentially methylated CpG sites identified comparing α-like to intermediate PanNETs

File Name: Supplementary Data 8
Description: Differentially methylated CpG sites identified comparing intermediate to β-like PanNETs

File Name: Supplementary Data 9
Description: Clinico-pathological and molecular characteristics of Chan et al. cohort
