# Supplementary material for: Epigenetic landscape of pancreatic neuroendocrine tumours reveals distinct cells of origin and means of tumour progression
Source: Commun Biol. 2020 Dec 7;3:740. doi: 10.1038/s42003-020-01479-y (PMC7721725; doi:10.1038/s42003-020-01479-y)
Supplement: Supplementary file 13 — Reporting summary [file 42003_2020_1479_MOESM13_ESM.pdf]

nature research

Corresponding author(s): Dr. Ilaria Marinoni

Last updated by author(s): Oct 23, 2020

Reporting Summary

Nature Research wishes to improve the reproducibility of the work that we publish. This form provides structure for consistency and transparency in reporting. For further information on Nature Research policies, see Authors & Referees and the Editorial Policy Checklist .

Statistics

For all statistical analyses, confirm that the following items are present in the figure legend, table legend, main text, or Methods section.

n/a	Confirmed	
☐	☒	The exact sample size (*n*) for each experimental group/condition, given as a discrete number and unit of measurement
☐	☒	A statement on whether measurements were taken from distinct samples or whether the same sample was measured repeatedly
☐	☒	The statistical test(s) used AND whether they are one- or two-sided<br>*Only common tests should be described solely by name; describe more complex techniques in the Methods section.*
☐	☒	A description of all covariates tested
☐	☒	A description of any assumptions or corrections, such as tests of normality and adjustment for multiple comparisons
☐	☒	A full description of the statistical parameters including central tendency (e.g. means) or other basic estimates (e.g. regression coefficient) AND variation (e.g. standard deviation) or associated estimates of uncertainty (e.g. confidence intervals)
☐	☒	For null hypothesis testing, the test statistic (e.g. *F*, *t*, *r*) with confidence intervals, effect sizes, degrees of freedom and *P* value noted<br>*Give P values as exact values whenever suitable.*
☒	☐	For Bayesian analysis, information on the choice of priors and Markov chain Monte Carlo settings
☐	☒	For hierarchical and complex designs, identification of the appropriate level for tests and full reporting of outcomes
☐	☒	Estimates of effect sizes (e.g. Cohen's *d*, Pearson's *r*), indicating how they were calculated

Our web collection on statistics for biologists contains articles on many of the points above.

Software and code

Policy information about availability of computer code

Data collection	Infinium® HumanMethylation450 BeadChip for IDAT files. Torrent Suite 5.0 and IonReporter 5.10 for NGS data analysis.
Data analysis	R version 3.5.0; ChAMP (v2.12.4); ape (v5.3); ConsensusClusterPlus (v1.46.0); tsne (v0.1-3); RnBeads (v2.0.1); GenomicRanges (v1.34.0); conumee (v1.16.0); survminer (v0.4.6); survival (v3.1-7).

For manuscripts utilizing custom algorithms or software that are central to the research but not yet described in published literature, software must be made available to editors/reviewers. We strongly encourage code deposition in a community repository (e.g. GitHub). See the Nature Research guidelines for submitting code & software for further information.

Data

Policy information about availability of data

All manuscripts must include a data availability statement. This statement should provide the following information, where applicable:

- Accession codes, unique identifiers, or web links for publicly available datasets
- A list of figures that have associated raw data
- A description of any restrictions on data availability

The datasets generated during the current study (UB-UCL cohort) are available in the ArrayExpress repository (EMBL-EBI, https://www.ebi.ac.uk/arrayexpress/, accession number: E-MTAB-7924). The datasets analysed during the current study (ICGC cohort) are available in the ICGC repository (ICGC, https://icgc.org/, projects: PAEN-AU and PAEN-IT). The datasets of sorted normal hematopoietic cells are available in the GEO repository (Gene Expression Omnibus, http://www.ncbi.nlm.nih.gov/geo/, accession number: GSE35069). The datasets of sorted acinar, duct, alpha and beta pancreatic cells are available in the EGA repository (European Genome-Phenome Archive, https://ega-archive.org/, accession number: EGAS00001002533). The datasets of sorted normal pancreatic fibroblastic cells are available in the GEO repository (Gene Expression Omnibus, http://www.ncbi.nlm.nih.gov/geo/, accession number: GSE80369). Chan et al. dataset is available in the GEO repository (Gene Expression Omnibus, http://www.ncbi.nlm.nih.gov/geo/, accession number: GSE117852).

Field-specific reporting

Please select the one below that is the best fit for your research. If you are not sure, read the appropriate sections before making your selection.

☒ Life sciences ☐ Behavioural & social sciences ☐ Ecological, evolutionary & environmental sciences

For a reference copy of the document with all sections, see nature.com/documents/nr-reporting-summary-flat.pdf

Life sciences study design

All studies must disclose on these points even when the disclosure is negative.

Sample size	We did not perform sample size calculation. We used the largest combined tumor sample and clinical datasets available to us.
Data exclusions	Samples for which tissue material was not available or did not reach sufficient tumor purity (>70%) were excluded.
Replication	Attempts of replication are described in the manuscript.
Randomization	Randomization is not applicable as no experimental groups were used in our study.
Blinding	Blinding is not applicable as no experimental groups were used in our study.

Reporting for specific materials, systems and methods

We require information from authors about some types of materials, experimental systems and methods used in many studies. Here, indicate whether each material, system or method listed is relevant to your study. If you are not sure if a list item applies to your research, read the appropriate section before selecting a response.

Materials & experimental systems

n/a	Involved in the study	
☐	☒	Antibodies
☒	☐	Eukaryotic cell lines
☒	☐	Palaeontology
☒	☐	Animals and other organisms
☐	☒	Human research participants
☒	☐	Clinical data

Methods

n/a	Involved in the study	
☒	☐	ChIP-seq
☒	☐	Flow cytometry
☒	☐	MRI-based neuroimaging

Antibodies

Antibodies used	DAXX (Sigma Aldrich, polyclonal rabbit), ATRX (Sigma Aldrich, polyclonal rabbit), Insulin (Sigma Aldrich, mouse, I-2018), Glucagon (Sigma Aldrich, mouse, G-2654), ARX (R&D Systems, sheep, AF7068), PDX1 (R&D Systems, mouse, MAB2419). All antibodies with human reactivity.
Validation	All antibodies are validated for immunohistochemistry application as stated on the corresponding Manufacturer's website. We further validated the performance in normal pancreatic islets (described in the manuscript).

Human research participants

Policy information about studies involving human research participants

Population characteristics	Relevant information on human research participants is provided in supplementary tables (1, 5 and 9) and figure 1A.
Recruitment	All patients that underwent surgery of a primary PanNET in the hospitals involved in the study and for which FFPE tissue material was available. Patients with DNA methylation data public available, involved in previous studies.
Ethics oversight	Bern: number 105/2015; London: number 09/H0722/27

Note that full information on the approval of the study protocol must also be provided in the manuscript.
